# Salidroside targets the Notch1/Hes5 axis to reconstruct the molecular innate immune–vascular network and correlates with repair after ischemic stroke

**DOI:** 10.3389/fimmu.2026.1756559

**Published:** 2026-02-11

**Authors:** Jing Zeng, Xiaohua Huang, Jingyi Zeng, Qi Huang, Wenyi Song, Youfeng Xie, Guining Liang, Qingyan Wei, Yating Lan, Donghua Zou, Rongjie Li, Lian Gu

**Affiliations:** 1 Guangxi University of Chinese Medicine, Nanning, Guangxi, China; 2 Department of Neurology, Affiliated Hospital of Youjiang Medical University for Nationalities, Baise, Guangxi, China; 3 Guangxi Key Laboratory of Artificial Intelligence for Genetic Diseases of Long-dwelling Nationalities, Baise, Guangxi, China; 4 Department of Neurology, The Second Affiliated Hospital of Guangxi Medical University, Nanning, Guangxi, China; 5 Department of Geriatrics, The Fifth Affiliated Hospital of Guangxi Medical University, Nanning, Guangxi, China; 6 Department of Neurology, Ruikang Hospital Affiliated to Guangxi University of Chinese Medicine, Nanning, Guangxi, China

**Keywords:** Hes5, ischemic stroke, molecular innate immunity, Notch1, salidroside, traditional Chinese medicine

## Abstract

**Background:**

Ischemic stroke (IS) induces profound dysregulation of the neuro–molecular innate immune–vascular network, yet the molecular immune states and regulatory mechanisms of key cellular subpopulations remain insufficiently defined. Although traditional Chinese medicine (TCM) exhibits multi-target immunomodulatory potential, its cell-type and cell-state–specific actions within the ischemic brain microenvironment at single-cell resolution remain unclear.

**Methods:**

Single-cell RNA sequencing was used to construct a cellular atlas of the ischemic mouse brain, followed by integrative bioinformatic analyses to characterize innate immune–related neural cell subpopulations and their regulatory networks. Network pharmacology and molecular docking were applied to identify salidroside (SAL), a major active compound of *Rhodiola*, and predict its potential molecular targets. *In vivo* experiments were performed to validate cellular and molecular changes associated with SAL treatment.

**Results:**

In a mouse model of IS, ischemic injury induced pronounced imbalances across multiple immune and glial cell subpopulations. A transcriptionally defined Notch1^+^ Hes5^+^ astrocyte (ASC), enriched for progenitor-like and reparative gene signatures, was markedly reduced after ischemic injury, whereas reactive SerpinA3N^+^ ASC and pro-inflammatory Sell^+^ microglia (MG) were expanded. Additionally, alterations were observed in immune-regulatory cell populations, including Cxcl12^+^ endothelial cells (ECs) and Gpr34^+^ Ptgs1^+^ MG. *In vivo* validation showed that SAL treatment was associated with modulation of Notch1/Hes5 signaling in ASC, reduced reactive ASC features, and partial normalization of ECs alterations, accompanied by changes consistent with attenuated neuroimmune activation. These effects coincided with altered intercellular communication, particularly involving NOTCH signaling.

**Conclusions:**

This study provides single-cell–level insights into innate immune microenvironment remodeling following IS and identifies a Notch1^+^ Hes5^+^ ASC subpopulation with transcriptional features associated with reparative-related programs and responsiveness to SAL. The findings suggest that SAL-associated neuroprotection was accompanied by modulation of ASC states and immune–glial communication, highlighting the potential of SAL-associated immunoregulatory effects at the single-cell level in IS.

## 1 Introduction

Stroke causes approximately 5.5 million deaths each year and has become one of the leading causes of mortality and disability worldwide (1). With the progressive westernization of lifestyle, factors such as hyperlipidemia, smoking, physical inactivity, unhealthy diet, and excessive alcohol consumption continuously drive the rising global incidence of stroke, with an increasingly evident trend toward younger onset (2). Stroke is broadly classified into ischemic stroke (IS) and hemorrhagic stroke (HS), with IS being the most prevalent subtype. IS was triggered by vascular occlusion in the brain, initiating a cascade of pathological events, including inflammation, oxidative stress, and disruption of the blood–brain barrier (BBB) (3, 4). Despite advances in reperfusion therapies, their clinical applicability remains limited due to strict indications and substantial risks, leaving effective interventions for IS still inadequate (5). A key challenge in current stroke research lies in elucidating the roles of diverse brain cell types during injury progression, particularly the immune cell driven cascades of inflammation and repair.

Numerous studies have demonstrated that following acute IS, peripherally infiltrating immune cells and central resident immune cells collectively orchestrate endothelial cells (ECs) activity via an intricate intercellular signaling network, thereby mediating both disruption and restoration of the BBB. This dual functionality may either exacerbate injury or facilitate repair (6, 7). Recent systematic reviews have further summarized the multidimensional features of this immune network: immune cells exhibit stage-dependent and specialized functions during ischemic progression, and their associated signaling pathways represent potential therapeutic targets (8, 9). Traditional Chinese medicine (TCM) has demonstrated multi-target therapeutic potential and has been reported to influence immune cell subpopulations, which may contribute to inflammation control in the early phase and tissue repair during later stages (10, 11). Furthermore, the bidirectional regulatory roles of glial cells have increasingly placed them at the center of anti-stroke therapeutic research (12).

At the molecular level, microglia (MG) and astrocytes (ASC) orchestrate the amplification of neuroinflammation through phenotype switching, inflammatory cytokine secretion, metabolic reprogramming, and intercellular communication, and they jointly contribute to tissue repair during the recovery phase (13). For example, ASC exhibit both neuroprotective and neurotoxic functions (14), while MG, the resident macrophages of the central nervous system (CNS), are key regulators of inflammatory responses and immune modulation in IS (15). Multiple studies have elucidated the crucial neuroprotective roles of ASC after ischemic injury. A comprehensive review highlighted that ASC contribute to neuroprotection through reactive astrogliosis and glial scar formation, promotion of neurogenesis, phagocytic clearance, and induction of ischemic tolerance, offering new cell-targeted therapeutic strategies for stroke (14). Mechanistic studies further revealed that the tryptophan metabolite 3-HKA suppresses AIM2 inflammasome activation in ASC, promoting their polarization toward the neuroprotective A2 phenotype while inhibiting the neurotoxic A1 phenotype. This shift is associated with vascular remodeling and BBB repair, ultimately improving long-term functional recovery (16). In addition, ECs have been shown to release microvesicles containing the neuronal transcription factor Ascl1 after injury, inducing ASC-to-neural progenitor trans-differentiation, thereby enhancing neuroplasticity and facilitating behavioral recovery revealing a novel vascular–neural regeneration mechanism (17). Notably, exogenous regulation can also modulate this immune network; for example, cottonseed oil reduces MG and astrocytic pro-inflammatory activation by inhibiting TLR4/NF-κB signaling, thereby lowering the proportion of neurotoxic A1 ASC and mitigating ischemic injury (18). However, the transcriptional remodeling, subpopulation heterogeneity, and precise regulatory nodes of these key cellular groups after IS remain insufficiently characterized, limiting the development of targeted therapies for the immune–glial network.

Single-cell RNA sequencing (scRNA-seq) provides unprecedented resolution for deciphering cellular composition and molecular landscapes in CNS diseases, enabling unbiased identification of novel cell states and functional markers (19). Meanwhile, TCM holds broad therapeutic promise for IS due to its multi-component and multi-pathway pharmacological nature. Compounds such as *Rhodiola*, astragaloside IV, and ginsenosides have been shown to exert neuroprotective functions through anti-inflammatory, anti-thrombotic, antioxidant mechanisms, and by regulating neural stem cell proliferation and differentiation (20–23). Nonetheless, the precise mechanisms by which TCM components act on specific immune–glial cell subpopulations remain unclear, and predictions of cell type and cell state–specific targets are lacking. Network pharmacology provides a systematic framework for elucidating the action networks of multi-component therapeutics (24). Integrating this approach with high-resolution scRNA-seq data may offer a comprehensive understanding of the cell type–specific molecular landscape in IS, identify novel functional cell subpopulations and regulatory hubs, and predict potential therapeutic targets modulated by TCM.

Thus, this study aims to analyze the cell-type-specific molecular immune landscape of IS by integrating scRNA-seq data from mouse models. The objective is to identify key cell subpopulations and their regulatory networks, ultimately identifying potential therapeutic targets for TCM-derived compounds. This approach will contribute to the development of novel neuroprotective strategies for IS treatment.

## 2 Materials and methods

### 2.1 Source of data

The gene expression data used in this study to investigate cellular heterogeneity and associated changes in IS were obtained from the Gene Expression Omnibus (GEO) database (25) (https://www.ncbi.nlm.nih.gov/geo/). The GSE171169 dataset contains brain tissue samples from four mice subjected to middle cerebral artery occlusion (MCAO) surgery on the platform GPL17021, representing the IS group (26). The GSE174574 dataset includes brain tissue samples from three mice in the MCAO group and three mice in the sham−surgery group on the platform GPL21103, with the sham group serving as the control (27). All single−cell data were processed according to the standard 10X Genomics workflow, involving cDNA fragmentation, amplification, library purification, quantification, and sequencing on the Illumina HiSeq platform. A total of seven MCAO and three sham−surgery mouse brain tissue samples were included in the study.

### 2.2 Data preprocessing and construction of single-cell atlases

This study integrated scRNA-seq data from two public databases generated using the 10x Genomics platform, initially yielding 69,446 cells. To minimize technical batch effects between the datasets, strict quality filtering was performed prior to downstream integration. Based on the Seurat package (28), quality control criteria included the total number of genes detected per cell (nFeature), total gene expression per cell (nCount), and the percentage of mitochondrial genes. Cells with nFeature values falling within the highest and lowest 1% intervals were filtered out, and cells with mitochondrial gene content exceeding 10% were excluded. Subsequently, the SCTransform method implemented in Seurat was employed for variance-stabilizing transformation using default parameter settings. This step concurrently accomplished library size normalization, selection of highly variable genes, and data scaling, thereby reducing technical variation arising from sequencing depth. Next, the Harmony algorithm (29) was applied to the SCTransform-processed data to correct for residual batch effects originating from the different datasets. Batch correction performance was assessed by visual inspection of UMAP embeddings colored by dataset origin, confirming effective mixing of cells from different datasets within major cell types. Ultimately, 66,862 high-quality cells were retained for subsequent analyses.

Cell clustering was performed using Seurat, and dimensionality reduction was conducted with the Uniform manifold approximation and projection (UMAP) algorithm (30) to construct a two-dimensional single-cell transcriptomic atlas. Cluster-specific marker genes were identified using the FindAllMarkers function, and cell types were annotated by manually integrating these markers with previously reported cell-type signatures from the literature.

### 2.3 Building pharmacological networks based on potential targets

Active compounds from TCM were retrieved from the traditional Chinese medicine systems pharmacology (TCMSP) database (https://www.tcmsp-e.com/) and screened based on oral bioavailability (OB ≥ 30%) and drug-likeness (DL ≥ 0.18) (31). Core targets and related pharmacological pathways were identified, and a pharmacological network linking TCM active compounds to IS targets was constructed using Cytoscape3.7.2 (32) software (https://cytoscape.org/).

### 2.4 Functional enrichment analysis

Pathway enrichment analysis was performed to identify metabolic or signaling pathways enriched within each cellular subpopulation. The Kyoto encyclopedia of genes and genomes (KEGG) pathway enrichment analysis (33) was performed on cluster marker genes using the ClusterProfiler package to explore the biological functions of each cells subpopulations. Statistical significance was set at *p* < 0.05.

For Gene set enrichment analysis (GSEA), genes were ranked based on the logarithmic fold changes in expression between treatment groups to determine whether gene expression changes associated with specific biological functions exhibited a non-random distribution, and the normalized enrichment score (NES) was calculated. The analysis of gene expression and phenotypic profiles across different cell subpopulations was aided by the MsigDB V7.4 database (c5.bp.v7.0.entrez.gmt and c2.cp.kegg.v7.0.symbols.gmt) (https://ngdc.cncb.ac.cn/databasecommons/database/id/1077). Within the context of GSEA, the NES serves as a key metric for assessing the enrichment degree of a gene set under specific phenotypic conditions. Positive and negative NES values indicate the activation (NES > 0) or inhibition (NES < 0) of signaling pathways, respectively, with statistical significance evaluated at *p* < 0.05.

### 2.5 Pseudotime analysis

Pseudotime analysis was performed using the Monocle3 package (34) to infer relative cellular state transitions associated with IS by modeling continuous changes in gene expression along putative transcriptional trajectories. Cells were first preprocessed and normalized using the preprocess_cds function, followed by dimensionality reduction via principal component analysis (PCA). The reduce_dimension function was subsequently applied to generate UMAP embeddings for visualization, and trajectory structures were learned using the learn_graph function. To define the directionality of pseudotime, the root state was determined based on a combination of prior biological knowledge and transcriptional characteristics. Specifically, we selected as the initiating subpopulations those that were relatively enriched under control conditions and exhibited transcriptional features consistent with homeostatic or early-stage states characterized by higher expression of canonical homeostatic markers and lower expression of activation-associated genes. Cells within these subpopulations were designated as root cells, and pseudotime values for all cells were calculated using the order_cells function. It should be noted that pseudotime ordering in this study reflects relative transcriptional progression rather than definitive lineage relationships or irreversible cell fate transitions. Trajectory structures and pseudotime distributions were visualized using the plot_cells function.

### 2.6 Gene regulatory network analysis

To identify key transcription factors (TFs) regulating each cell subpopulation, gene regulatory network (GRN) inference was performed using the pySCENIC package (35). TF binding motifs were retrieved from the JASPAR database (https://jaspar.genereg.net) to elucidate the underlying transcriptional regulatory programs.

### 2.7 Molecular docking validation

Molecular docking was performed using AutoDock (36) to validate interactions between core active compounds and their targets. Core compounds acted as ligands, and target genes from cell subpopulations acted as receptors. 3D structures of compounds and target genes were retrieved from PubChem (https://pubchem.ncbi.nlm.nih.gov/) and the Protein Data Bank (PDB) (https://www.rcsb.org/). Preprocessing steps included structure optimization, hydrogenation, and ionization. Molecular docking methods were used to analyze receptor-ligand binding modes, and interaction energies were calculated to identify optimal binding complexes (37).

### 2.8 Animal model establishment, drug administration, and tissue processing

Six-week-old male C57BL/6 mice (weighing 18–25 g) were used in this study. After one week of acclimatization under standard conditions, the mice were randomly assigned to three groups (n=3 per group): a sham-operated group, a model group, and a SAL-treated group (50 mg/kg). The transient MCAO model was established using the intraluminal filament method. Briefly, mice were anesthetized via intraperitoneal injection of 0.5% pentobarbital sodium (45 mg/kg). Following isolation of the left common carotid artery, external carotid artery, and internal carotid artery, a silicon-coated nylon monofilament (diameter 0.20-0.25 mm) was inserted through the stump of the external carotid artery and advanced to the origin of the middle cerebral artery (insertion depth approximately 1.6-1.9 cm from the bifurcation) to occlude blood flow. After 2 hours of ischemia, the filament was gently withdrawn to allow reperfusion. The sham-operated group underwent the same surgical procedure except for filament insertion. SAL was administered therapeutically rather than prophylactically. The SAL-treated group received intraperitoneal administration of SAL (50 mg/kg, once daily for 3 consecutive days) starting 2 hours after reperfusion, while the model and sham-operated groups received an equivalent volume of normal saline at the same time points. After the final administration, mice were subjected to cardiac perfusion with ice-cold PBS and 4% paraformaldehyde (PFA). Brains were harvested, fixed, paraffin-embedded, and coronally sectioned at a thickness of 4 μm for subsequent analysis. The selected dose was based on prior studies demonstrating neuroprotective efficacy and acceptable pharmacokinetic profiles of SAL in rodent models of cerebral ischemia.

### 2.9 Immunohistochemical and immunofluorescent quantification of key cellular subpopulations

To evaluate changes in specific cellular subpopulations within the brain, immunofluorescence staining was performed on sections obtained from the ipsilateral cortex and basal ganglia. At the experimental endpoint, mice were transcardially perfused with 4% paraformaldehyde, and brains were collected. Coronal sections were prepared spanning Bregma +1.0 mm to −1.0 mm. Paraffin-embedded tissues were sectioned at 5 μm and processed using standard deparaffinization (with Solarbio Eco-friendly Dewaxing Solution, YA0031), rehydration, and antigen retrieval procedures (using Servicebio 20× Tris-EDTA Antigen Retrieval Buffer (pH 9.0), Cat# G1203-250ml, diluted according to the manufacturer’s instructions). Frozen sections were cut at 10 μm, equilibrated at room temperature, and directly subjected to blocking. Following blocking, tissue sections were incubated with primary antibodies specific to the target cell populations (Notch1, Proteintech, 10062-2-AP, 1:300; Hes5, Proteintech, 22666-1-AP, 1:100; GFAP, Servicebio, GB11096-50, 1:1000; SerpinA3 (AACT), Zenbio, 680005, 1:300; Cxcl12, Proteintech, 17402-1-AP, 1:400; Flt1 (VEGFR1), ABclonal, A0058, 1:100) at 4 °C overnight. The next day, sections were thoroughly washed with phosphate-buffered saline (PBS) and then incubated with the corresponding fluorophore-conjugated secondary antibodies. Nuclear counterstaining was performed using 4’,6-Diamidino-2-phenylindole (DAPI). After staining, sections were mounted with antifade medium and protected from light exposure. Images were acquired using a confocal fluorescence microscope. Multiple fields within the ischemic penumbra were randomly selected for imaging. All imaging parameters were kept constant across animals and experimental groups to ensure comparability. Image analysis was conducted using ImageJ software. Positive cells were quantified by applying a uniform threshold across samples, and cell density or proportional abundance within the selected fields was calculated for subsequent statistical analysis.

### 2.10 Statistical analysis

All the biological information analysis in this study were conducted based on Bioinforcloud platform (http://www.bioinforcloud.com/). For comparisons of cell subpopulation abundances between experimental groups, statistical testing was performed at the biological replicate level rather than at the single-cell level to avoid pseudo-replication. Cell proportions were first calculated for each individual sample, followed by group-wise statistical comparison. Statistical significance was defined as *p* < 0.05.

## 3 Results

### 3.1 Single-cell atlas of IS

**Figure 1:**
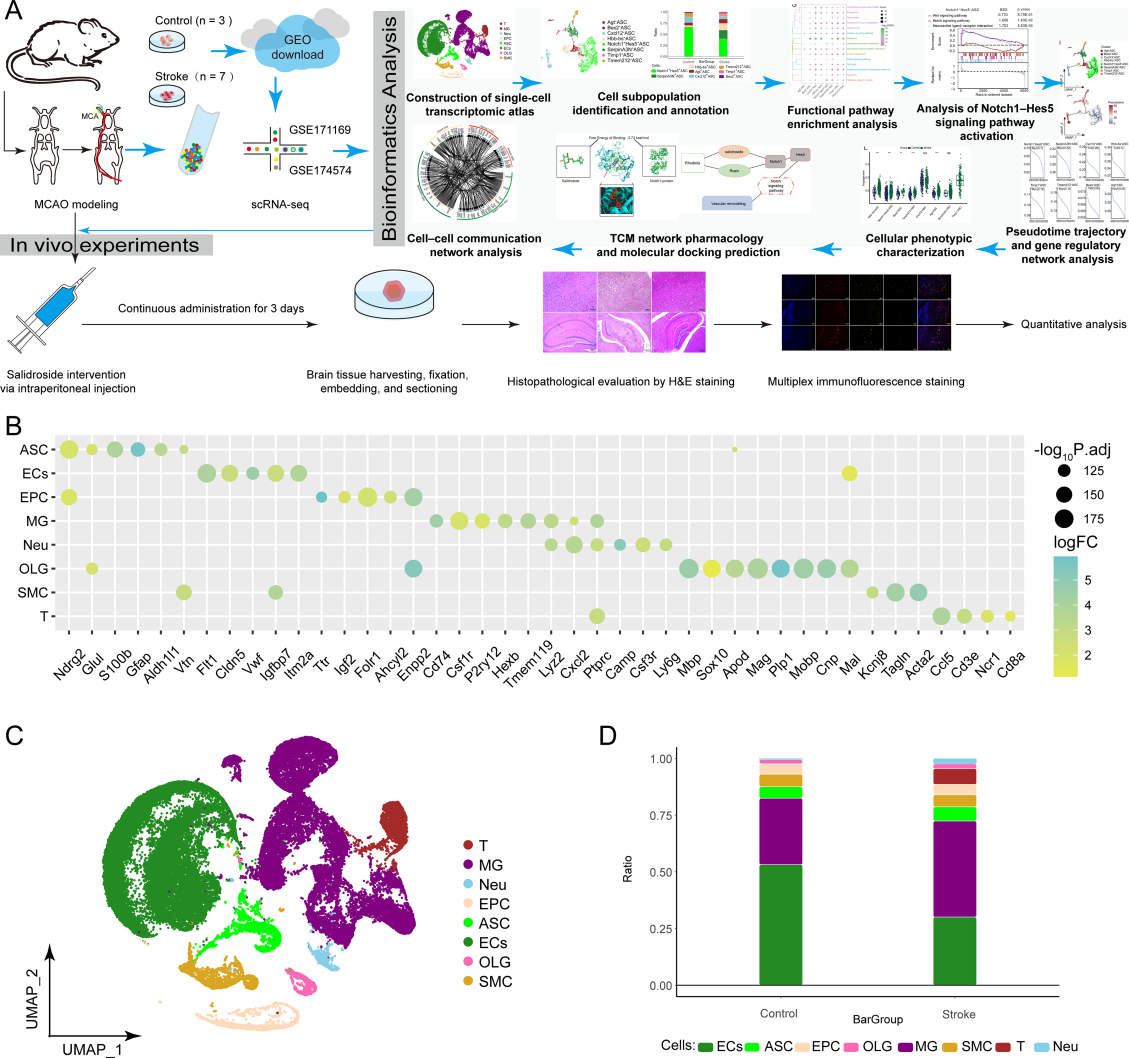
Single cell landscape of ischemic stroke (IS). Single-cell landscape of the Middle cerebral artery occlusion (MCAO) mouse model: This study analyzed single-cell RNA sequencing (scRNA-seq) data (10X Genomics) from both the control and stroke groups, following a comprehensive workflow. **(A)** Schematic diagram of the overall workflow for a single-cell transcriptomic study in a mouse model of IS. **(B)** Bubble plots showing specific marker genes in different cell types. **(C)** Eight identified major cell types, namely T cells (T), microglial (MG), neutrophils (Neu), ependymocytes (EPC), astrocyte (ASC), endothelial cell (ECs), oligodendrocyte (OLG), and smooth muscle cell (SMC), were used. **(D)** Single-cell ecological component ratio bar graph demonstrating the differences in composition between cell clusters. MCAO, middle cerebral occlusion; T, T cells; MG, microglial cells; Neu, neutrophils; EPC, ependymocytes; ASC, astrocytes; ECs, endothelial cells; OLG, oligodendrocytes; SMC, smooth muscle cells.

To elucidate the roles of distinct cell types in the pathophysiological mechanisms of IS, we performed scRNA-seq analysis of brain tissues obtained from a mouse MCAO model, with the overall workflow illustrated in **Figure 1A**. Through unsupervised clustering, a total of eight major cell types were identified from 66,862 cells, including astrocytes (ASC), endothelial cells (ECs), ependymocytes (EPC), microglia (MG), T cells (T), oligodendrocytes (OLG), neutrophils (Neu), and smooth muscle cells (SMC) (**Figure 1C**). Cell-type annotation was based on the expression patterns of established cell-type–specific marker genes (**Figure 1B**). Comparative analysis further revealed the cellular abundance proportions at the single-cell level in brain tissue after IS, with ECs constituting the largest proportion in the control group, whereas MG emerged as the predominant cell population in the Stroke group (**Figure 1D**). Collectively, this transcriptomic atlas demonstrates cell-type–specific alterations in cellular composition within the ischemic brain injury microenvironment.

### 3.2 ASC subpopulations exhibit distinct neuroimmune functions

**Figure 2:**
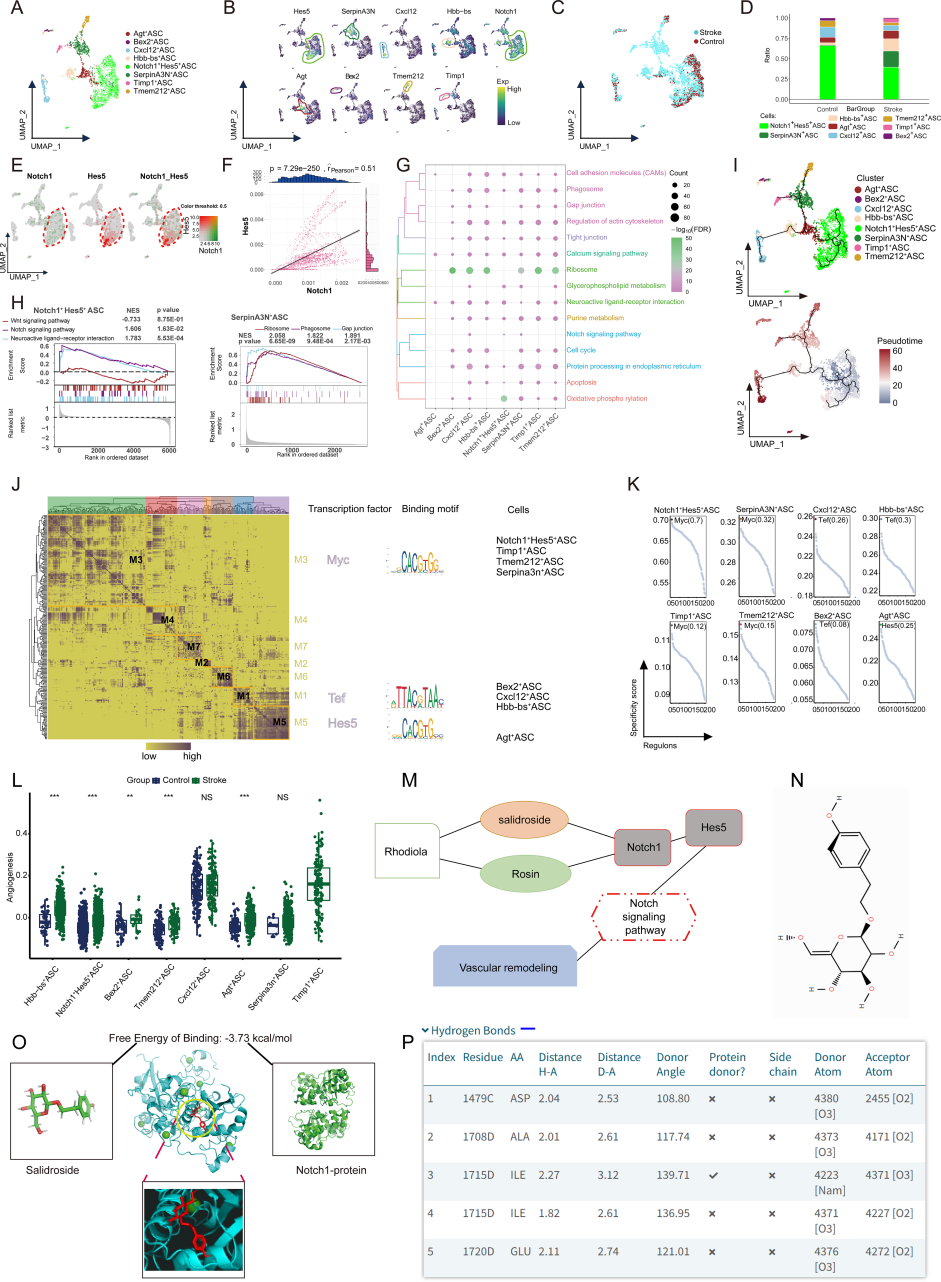
Single-cell landscape of ASC populations. **(A)** Identification of ASC in IS with single-cell mapping showing clusters of cells in ASC. **(B)** Marker genes for each subpopulation of ASC. **(C)** Single-cell mapping showing ASC cell clusters in Control and Stroke group samples from mouse model. **(D)** Differential abundance of ASC subpopulations in IS cell clusters in the Stroke and Control groups. **(E)** Demonstration of Notch1 and Hes5 double-positive gene co-expression. **(F)** Correlation between Notch1 and Hes5 double-positive genes. **(G)** Biological pathway enrichment in specific clusters of ASC. **(H)** Gene set enrichment analysis (GSEA) results demonstrating biological pathway activation in Notch1^+^Hes5^+^ASC and SerpinA3N^+^ASC. **(I)** Pseudotime analysis. **(J)** Heatmap-Transcription factors (TF) binding sequence cell clusters demonstrating the gene regulatory network (GRN) of ASC clusters. **(K)** TFs regulating marker expression of ASC. **(L)** Scoring between ASC subpopulations and angiogenesis. **(M)** Pharmacological network of Salidroside (SAL), a traditional Chinese medicine (TCM) active agent for the treatment of cerebral IS. **(N)***Rhodiola rosea* acted mainly through SAL. **(O)** Docking model of SAL with Notch1 protein-binding molecule showed a free energy release of -3.73 kcal/mol. **(P)** The major binding sites of SAL with Notch1 included GLU-1720, ASP-1479, ALA-1708, and others. (Statistical significance notation: NS indicates no significance; ** represents p-value < 0.01; *** represents p-value < 0.001). ASC, astrocytes; GSEA, gene set enrichment analysis; IS, ischemic stroke; TF, transcription factor; SAL, salidroside; TCM, traditional Chinese medicine; GRN, gene regulatory network.

The ASC cell population was annotated, identifying eight distinct subpopulations (**Figure 2A**). These subpopulations were defined by their specific marker genes (such as Hes5, SerpinA3N, and Cxcl12) (**Figure 2B**), and their composition and distribution were shown in samples from both the control and stroke groups (**Figure 2C**). At the single-cell transcriptomic level, we observed that, compared with the control group, the relative abundance of the Notch1^+^ Hes5^+^ ASC subpopulation was markedly reduced in the stroke group, whereas the proportion of SerpinA3N^+^ ASC was increased (**Figure 2D**). Within the Notch1^+^ Hes5^+^ ASC subpopulation, Notch1 and Hes5 transcripts exhibited coordinated expression with a significant positive correlation (**Figures 2E, F**), suggesting transcriptional coupling within this subpopulation. Functional analysis revealed significant enrichment and activation of the Notch signaling pathway in Notch1^+^ Hes5^+^ ASC, and the phagocytic pathway in SerpinA3N^+^ ASC (*p* < 0.05) (**Figures 2G, H**). Pseudotime analysis suggested that Notch1^+^ Hes5^+^ ASC occupy an early position along the inferred differentiation trajectory of ASC (**Figure 2I**). Additionally, GRN analysis identified TFs such as Myc, Tef, and Hes5 that regulate specific gene expression in different modules (**Figures 2J, K**), indicating potential regulatory relationships underlying ASC transcriptional heterogeneity, thereby promoting cell differentiation and facilitating neuroprotection (13). Scoring analyses further revealed differential associations between ASC subpopulations and angiogenesis-related gene sets (**Figure 2L**). In combination with a comprehensive literature review, we correlated the single-cell transcriptomic findings with TCM targets. We constructed a pharmacological network for *Rhodiola rosea* in the treatment of IS (**Figure 2M**), where SAL is predicted to be associated with modulation of the Notch1-related signaling axis (**Figure 2N**). Molecular docking analysis revealed a binding free energy of -3.73 kcal/mol between SAL and Notch1 protein (**Figure 2O**), and key docking sites were identified (**Figure 2P**). These results indicate that Notch1^+^ Hes5^+^ ASC exhibits transcriptional and regulatory features that suggest potential relevance to therapeutic modulation in the context of TCM treatment for ischemic brain injury. The network pharmacology and docking results suggest a potential association between SAL and Notch signaling–related pathways, rather than establishing a direct causal mechanism.

### 3.3 ECs subpopulations display transcriptional signatures associated with post-ischemic inflammatory responses

**Figure 3:**
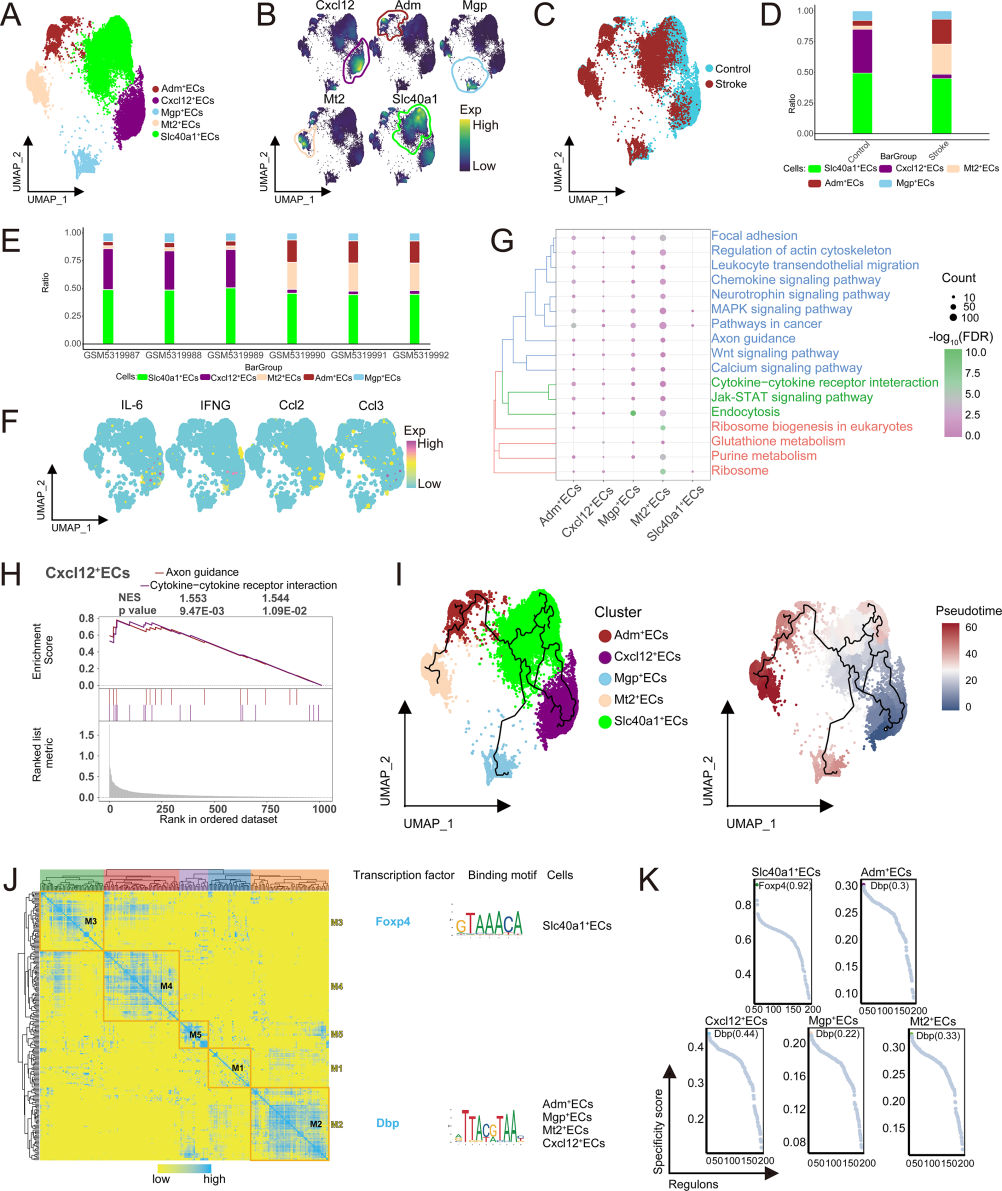
Single-cell landscape of ECs populations. **(A)** Identification of ECs in IS with single-cell mapping showing clusters of cells with ECs. **(B)** Marker genes for specific clusters of ECs. **(C)** Single-cell mapping showing cell clusters of ECs in Control and Stroke group samples from mouse model. **(D)** Differences in cellular abundance of ECs subpopulations in the Stroke and Control groups of ECs. **(E)** Abundance of ECs subpopulations in the samples. **(F)** Mapping of inflammatory factors ECs subpopulations. **(G)** Biological pathways in specific clusters of ECs. **(H)** Activation of biological pathways in EC-specific clusters. **(I)** Single-cell mapping of trajectories and pseudotime values of ECs progression. **(J)** Heatmap-TF binding sequence cell clusters demonstrating GRN in ECs clusters. **(K)** TFs regulate the expression of markers of ECs clusters. ECs, endothelial cells; IS, ischemic stroke; TF, transcription factor; GRN, gene regulatory network.

This study annotated ECs subpopulations using specific marker genes and identified five distinct ECs subpopulations (**Figures 3A, B**), and the composition and distribution of ECs subpopulations were shown in samples from both the control and stroke groups (**Figure 3C**). Compared to the control group, the abundance of Cxcl12^+^ ECs significantly decreased in the stroke group (**Figure 3D**), with notable differences in the abundance of subpopulations between individual samples (**Figure 3E**). Further analysis showed that Cxcl12^+^ ECs highly express inflammatory chemokines (Ccl2, Ccl3) and cytokines (IL-6, IFN-γ) (**Figure 3F**), and they are significantly enriched and activated in the cytokine-cytokine receptor interaction pathway (**Figures 3G, H**), suggesting that changes in the abundance of Cxcl12^+^ ECs are accompanied by altered inflammatory-related transcriptional signatures during ischemic conditions. Pseudotime analysis suggested that the Cxcl12^+^ ECs occupy an early position along the inferred ECs differentiation trajectory (**Figure 3I**). Furthermore, GRN analysis identified five regulatory modules (**Figure 3J**), revealing the role of TFs such as Foxp4 and Dbp in regulating specific gene expression (**Figure 3K**). In summary, Cxcl12^+^ ECs exhibit transcriptional signatures indicative of involvement in early inflammatory responses. Their post-ischemic reduction may reflect ECs exhaustion or phenotypic transition, a finding consistent with previously described transcriptional features of ECs dysfunction and immune cell recruitment.

### 3.4 MG subpopulations heterogeneity and functional changes in IS

**Figure 4:**
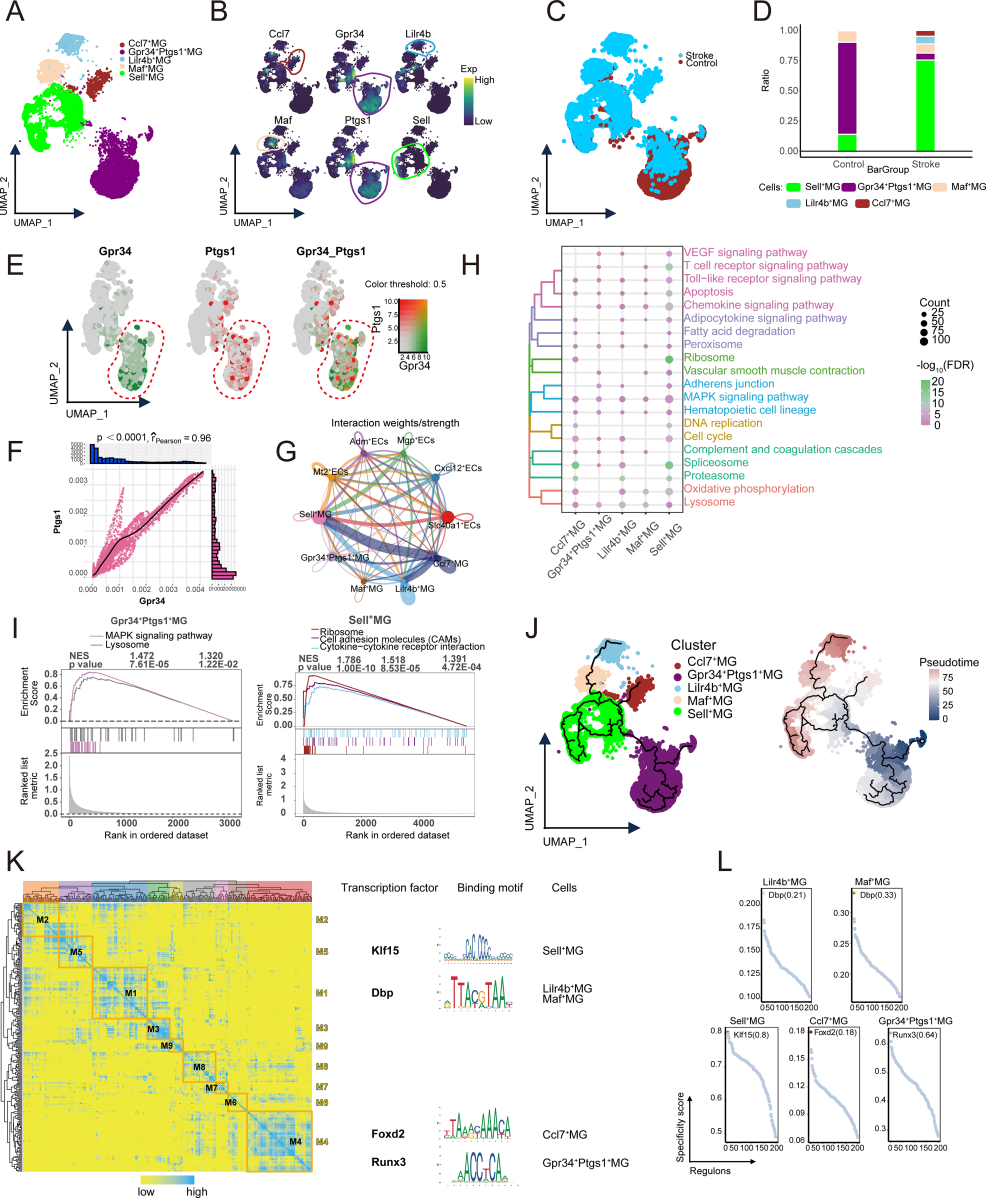
Single-cell landscape of MG clusters. **(A)** Identification of MG clusters in IS with single-cell mapping showing clusters of MG. **(B)** Marker genes for specific MG clusters. **(C)** Single-cell mapping revealed MG in the mouse model Control and Stroke groups. **(D)** Difference in cell abundance of MG in Stroke and Control groups. **(E)** Co-expression of Gpr34^+^ Ptgs1^+^MG double positive genes. **(F)** Correlation between Gpr34 and Ptgs1 double positive genes. **(G)** Communication between ECs and MG. **(H)** Biological pathways in MG-specific clusters. **(I)** Activation of biological pathways in MG-specific clusters. **(J)** Single-cell mapping of trajectories and pseudotime values of MG progression. **(K)** Heatmap-TF binding sequence cell clusters demonstrate GRN in MG clusters. **(L)** TFs regulate the expression of MG cluster markers. MG, microglial cells; ECs, endothelial cells; IS, ischemic stroke; TF, transcription factor.

Through clustering analysis and annotation based on specific marker genes, five distinct MG subpopulations were identified (**Figures 4A, B**), and their composition and distribution were shown in samples from both the control and stroke groups (**Figure 4C**). The analysis revealed that Gpr34^+^ Ptgs1^+^ MG dominated the control group but significantly decreased in the stroke group. In contrast, Sell^+^ MG were significantly more abundant in the stroke group (**Figure 4D**). Subsequent analysis showed that Gpr34 and Ptgs1 genes were co-expressed in MG, with a significant positive correlation (*p* < 0.0001) (**Figures 4E, F**). Cell communication analysis between ECs and MG (**Figure 4G**) indicated frequent communication between Gpr34^+^ Ptgs1^+^ MG and other ECs subpopulations. Functional analysis showed that Gpr34^+^ Ptgs1^+^ MG were enriched and activated in the lysosomal pathway, whereas Sell^+^ MG were enriched and activated in the cell adhesion molecule (CAMs) pathway (**Figures 4H, I**). Pseudotime analysis suggested that Gpr34^+^ Ptgs1^+^ MG occupy an early position along the inferred MG differentiation trajectory, with transitions toward other MG subpopulations (**Figure 4J**). GRN analysis identified nine regulatory modules, with TFs such as Klf15, Dbp, Foxd2, and Runx3 playing key roles in regulating gene expression (**Figures 4K, L**). These transcriptional differences suggest a shift from homeostatic-associated gene expression toward profiles enriched for adhesion-related pathways, rather than directly indicating functional impairment, collectively suggesting a transcriptional landscape consistent with an exacerbated inflammatory environment.

### 3.5 OLG subpopulations functional changes and repair potential after stroke

**Figure 5:**
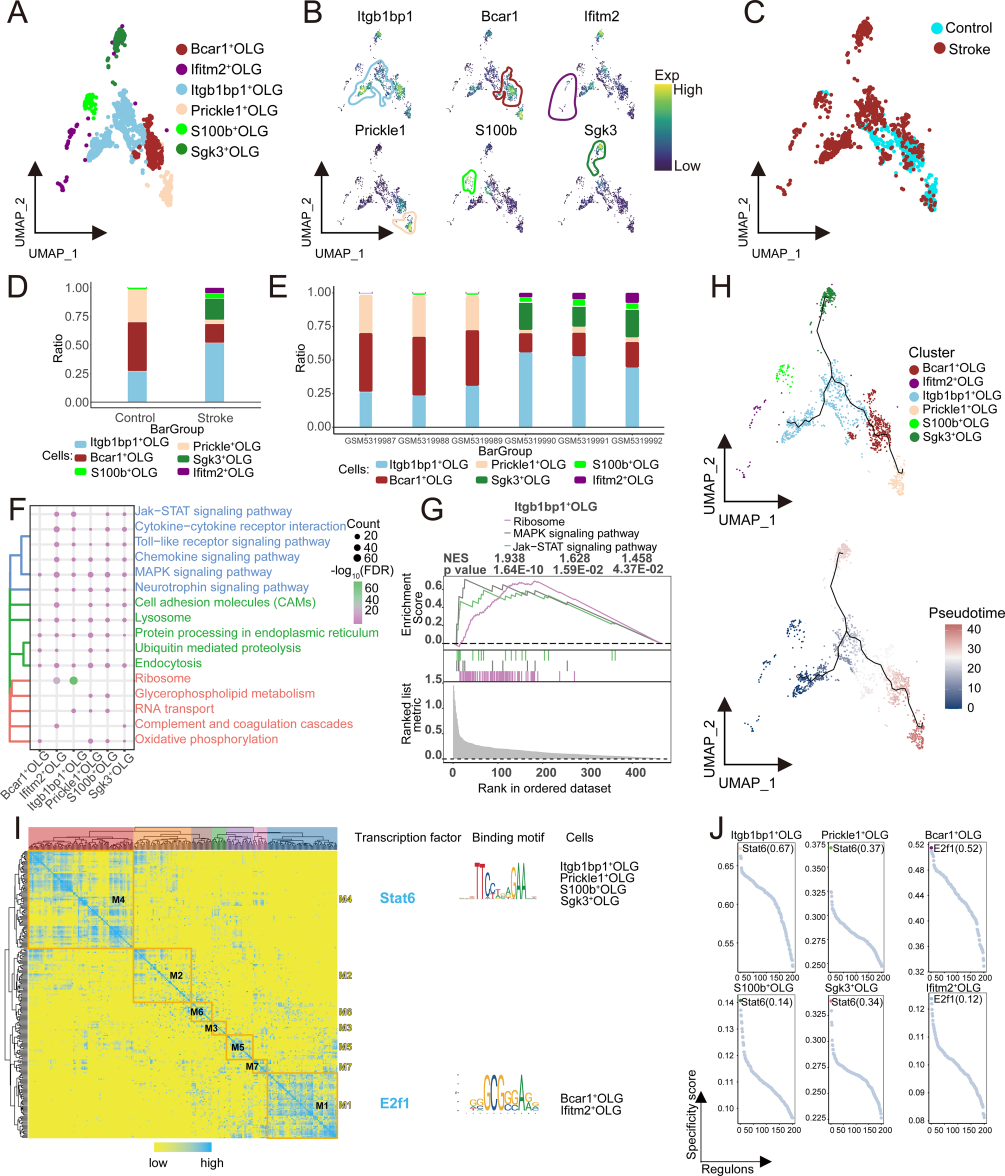
Single-cell landscape of OLG clusters. **(A)** Identification of OLG clusters in IS with single-cell mapping showing cellular clusters of OLG. **(B)** Marker genes for specific OLG clusters. **(C)** Single-cell mapping reveals the distribution and differences between control and stroke group samples in the mouse model. **(D)** Differences in OLG cluster abundance in the Stroke and Control groups. **(E)** Demonstration of the abundance of subpopulations in the samples. **(F)** Biological pathways in OLG-specific clusters. **(G)** Activation of biological pathways in OLG-specific clusters. **(H)** Single-cell mapping of trajectories and pseudotime values of OLG progression. **(I)** Heatmap-TF binding sequence cell clusters demonstrating GRN in OLG clusters. **(J)** TFs regulate the expression of OLG cluster markers. OLG, oligodendrocyte; IS, ischemic stroke; TF, transcription factor; GRN, gene regulatory network.

Through clustering analysis and annotation based on specific marker genes, six distinct OLG subpopulations were identified (**Figures 5A, B**), and their distribution in samples from the control and stroke groups was shown (**Figure 5C**). The analysis found that the abundance of Itgb1bp1^+^ OLG was significantly increased in the stroke group (**Figure 5D**), with notable differences in the abundance across different samples (**Figure 5E**). Functional analysis showed that Itgb1bp1^+^ OLG were significantly enriched and activated in ribosome, JAK-STAT, and MAPK signaling pathways (**Figures 5F, G**). Pseudotime analysis suggested that Itgb1bp1^+^ OLG occupy an early position along the inferred OLG differentiation trajectory, followed by transitions toward other OLG subpopulations (**Figure 5H**). Additionally, GRN analysis identified seven regulatory modules, with TFs such as Stat6 and E2f1 regulating specific gene expression (**Figures 5I, J**). Overall, Itgb1bp1^+^ OLG responds to inflammation and repair signals after stroke via MAPK and JAK-STAT pathways. These pathways are closely related to OLG proliferation and differentiation. Furthermore, they participate in axonal regeneration and myelin repair, potentially contributing to processes associated with post-stroke brain tissue repair and recovery.

### 3.6 SMC subpopulations changes and their transcriptional alterations associated with in post-stroke vascular dysfunction

**Figure 6:**
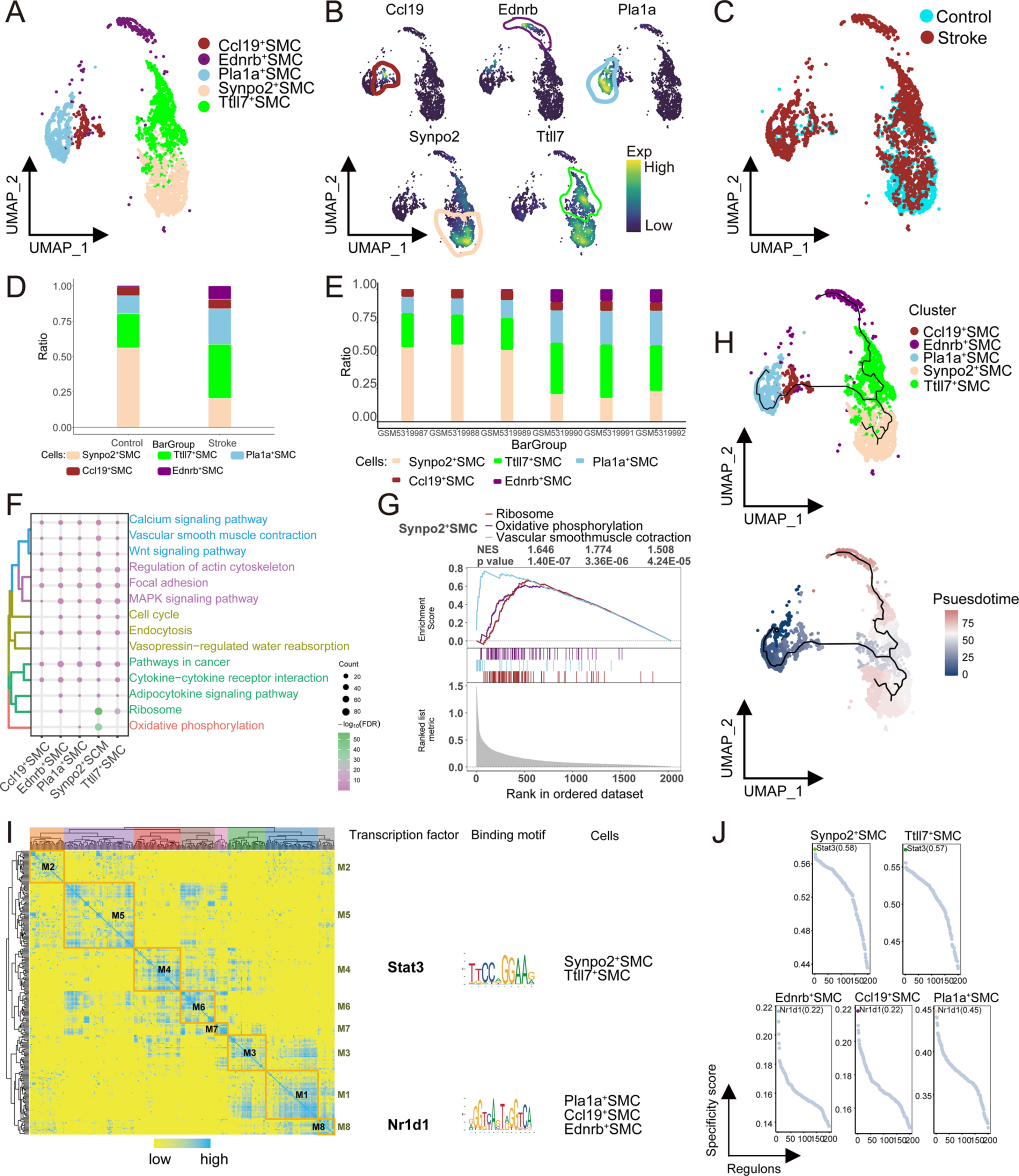
Single-cell landscape of SMC clusters. **(A)** Identification of SMC clusters in IS with single-cell mapping showing clusters of SMC cells. **(B)** Marker genes for specific SMC clusters. **(C)** Single-cell atlas reveals the distribution of samples from the control and stroke groups in the mouse model. **(D)** Differences in cell abundance between SMC clusters in the Stroke and Control groups. **(E)** Demonstrating the abundance of subpopulations in the samples. **(F)** Biological pathways in SMC-specific clusters. **(G)** Activation of biological pathways in SMC-specific clusters. **(H)** Single-cell mapping of SMC progression trajectories and pseudotime values. **(I)** Heatmap-TF binding sequence cell clusters demonstrate GRN in SMC clusters. **(J)** TFs regulate the expression of SMC cluster markers. SMC, smooth muscle cells; IS, ischemic stroke; TF, transcription factor; GRN, gene regulatory network.

Through clustering analysis and annotation based on specific marker genes, five distinct SMC subpopulations were identified (**Figures 6A, B**), and their distribution in samples from the control and stroke groups was shown (**Figure 6C**). The analysis revealed that Synpo2^+^ SMC were the most abundant in the control group but exhibited a marked reduction in abundance in the stroke group (**Figure 6D**), with notable differences in the abundance of subpopulations across different samples (**Figure 6E**). We also explored the biological functions of each SMC subpopulation. SMC were related to cellular metabolism and motility, with Synpo2^+^ SMC closely associated with vascular smooth muscle contraction and oxidative phosphorylation (**Figure 6F**). GSEA showed that Synpo2^+^ SMC were significantly activated in vascular smooth muscle contraction (*p* < 0.05) and oxidative phosphorylation (*p* < 0.05) pathways (**Figure 6G**). The pseudotime analysis suggested that Pla1a^+^ SMC reside at an early stage of the inferred differentiation trajectory, potentially giving rise to subsequent subpopulations. Among these, Synpo2^+^ SMC appeared as a likely terminal point in one branch of the trajectory (**Figure 6H**). Additionally, GRN analysis identified eight regulatory modules, with TFs such as Stat3 and Nr1d1 regulating specific gene expression (**Figures 6I, J**). In summary, Synpo2^+^ SMC likely play a key role in the vascular smooth muscle contraction pathway. The significant reduction of Synpo2^+^ SMC in the stroke group may reflect transcriptional features associated with vascular dysfunction and impaired contractile capacity, consistent with dysregulated smooth muscle contraction.

### 3.7 Cell–cell communication network and the regulatory effects associated with SAL treatment on key cell subpopulations

**Figure 7:**
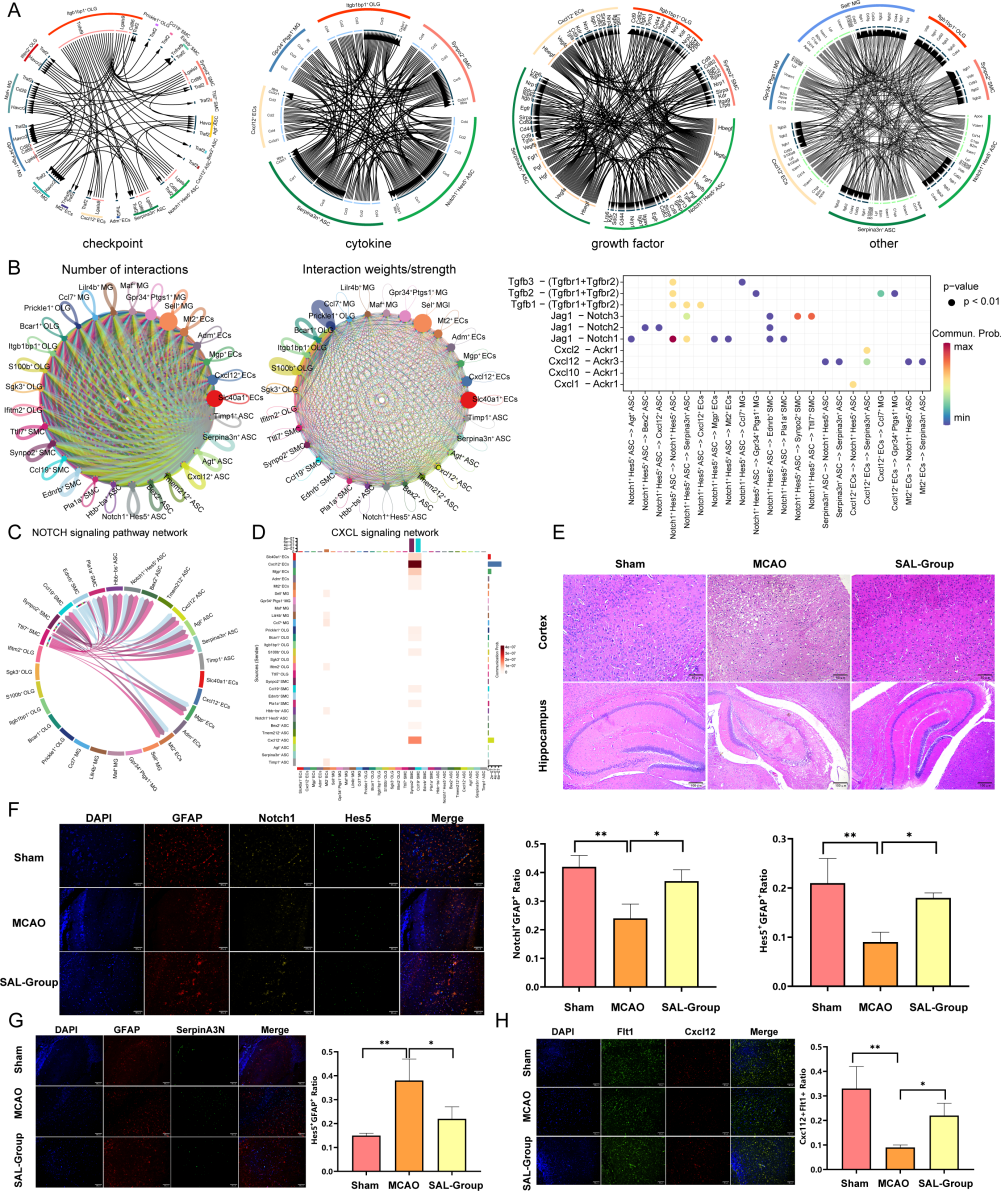
Intercellular communication networks after ischemia and the effects of SAL on key cell subpopulations. **(A)** CellChat-based analysis depicting intercellular interactions mediated by checkpoint, cytokine, growth factor, and other ligand–receptor signaling in ischemic brain tissue. **(B)** Left: Interaction patterns between the Notch1^+^ Hes5^+^ ASC subpopulation and microenvironmental cells. Right: Communication probabilities and significance of representative ligand–receptor pairs within the TGFβ, CXCL, and NOTCH pathways. **(C)** Chord diagram showing interaction strength within the NOTCH signaling pathway across different cell populations. **(D)** Heatmap illustrating communication intensity within the CXCL signaling pathway among various cell types. **(E)** Hematoxylin eosin staining of cortical and hippocampal sections from mouse brain tissue, showing histopathological alterations. **(F)** Immunofluorescence staining and quantitative analysis of Notch1^+^ Hes5^+^ ASC (Notch1+/Hes5+/GFAP+). **(G)** Immunofluorescence staining and quantitative analysis of SerpinA3N^+^ ASC (SerpinA3N+/GFAP+). **(H)** Immunofluorescence staining and quantitative analysis of Cxcl12^+^ ECs (Cxcl12+/Flt1+). (Statistical significance notation: * represents p-value < 0.05; ** represents p-value < 0.01). ASC, astrocytes; ECs, endothelial cells.

To investigate the impact of SAL on critical cell subpopulations following IS, we first constructed a cell–cell communication network using CellChat analysis. The results revealed inferred increases in ligand–receptor communication probabilities across checkpoint, cytokine, growth factor, and other ligand–receptor mediated communication pathways, with particularly prominent alterations involving Notch1^+^ Hes5^+^ ASC, SerpinA3N^+^ ASC, and Cxcl12^+^ ECs (**Figure 7A**). Cell–cell communication probabilities and statistical significance of ligand–receptor interactions were inferred using CellChat built-in permutation framework, with interaction strength quantified as communication probability scores. Furthermore, we systematically evaluated the interaction patterns between Notch1^+^ Hes5^+^ ASC cells and microenvironmental cells, which revealed the breadth and connection density of subpopulation communication, thereby identifying their role as an active signaling hub. In addition, analysis of key ligand–receptor pairs specifically highlighted the communication probabilities and statistical significance of representative interactions within the TGFβ, CXCL, and NOTCH pathways (**Figure 7B**). Further pathway-focused analysis demonstrated a substantial increase in both upstream and downstream signaling exchanges within the NOTCH pathway (**Figure 7C**). In the CXCL signaling axis, communication intensity was strongly driven by chemokine-mediated interactions (**Figure 7D**). Based on the key cell subpopulations identified through the communication analysis, we performed *in vivo* validation using a mouse ischemic model to examine the regulatory effects of SAL. Histological assessments revealed significant structural damage in the cortex and hippocampus of MCAO model mice, while the SAL-treated group exhibited more preserved brain tissue architecture and reduced injury severity (**Figure 7E**). At the cellular level, immunofluorescence analysis showed that compared to the Sham group, the model group displayed reduced co-expression levels of Notch1/GFAP and Hes5/GFAP, and these changes were markedly reversed by SAL treatment (**Figure 7F**). Similarly, compared to the Sham group, SerpinA3N^+^ expression was substantially upregulated in the model group, while SAL administration significantly reduced the SerpinA3N/GFAP ratio (**Figure 7G**), consistent with our previous single-cell analysis results. Furthermore, the experiment also revealed that Cxcl12 expression was significantly reduced in the model group but showed notable improvement following SAL intervention (**Figure 7H**). Collectively, these results indicate that SAL treatment was associated with a partial recovery of the Notch1^+^ Hes5^+^ ASC subpopulation, attenuation of aberrant activation in selected cell subpopulations, and modulation of intercellular communication networks following ischemic injury, thereby reshaping the post-ischemic microenvironment in a manner consistent with neuroprotective effects.

## 4 Discussion

This study constructed a comprehensive single-cell atlas of IS, delineating the remodeling of the brain’s cellular ecosystem. We identified significant shifts in cellular subpopulations post-ischemia, the expansion of pro-inflammatory or reactive states, such as SerpinA3N^+^ ASC, Sell^+^ MG, and Itgb1bp1^+^ OLG, alongside the contraction of potentially protective or homeostatic populations, including Notch1^+^ Hes5^+^ ASC, Cxcl12^+^ EC, Gpr34^+^ Ptgs1^+^ MG, and Synpo2^+^ SMC. These changes in cellular abundance are consistent with a fundamental reprogramming of neuro-molecular innate immune and vascular functions. Furthermore, the findings suggest that the key Notch1^+^ Hes5^+^ ASC subpopulation may represent a putative ASC state linked to repair-associated transcriptional programs, and this subpopulation may be responsive to SAL-associated modulation.

Our findings highlight cellular changes that underscore the pivotal involvement of non-neuronal cells in post-stroke pathology and repair. A central discovery was the significant reduction of the Notch1^+^ Hes5^+^ ASC subpopulation. While this subpopulation was diminished in abundance, its associated Notch signaling pathway was functionally critical. This was consistent with the established role of Hes family TFs in vascular development and remodeling (38). Given the putative upstream positioning of Notch1^+^ Hes5^+^ ASC in neurovascular-support programs, their reduction coincides with diminished Notch1-mediated transcriptional activation, which is implicated in maintaining ECs stability, regulating glial–vascular communication, and coordinating reparative responses. The loss of Notch1^+^ Hes5^+^ ASC may therefore reflect disruption of a developmental signaling–associated program that is relevant to repair-related processes in the brain, and may be associated with altered vascular–immune crosstalk and tissue reconstruction. Conversely, the expansion of SerpinA3N^+^ ASC signifies a shift toward a reactive astroglial state. Although SerpinA3N has been reported to exert anti-inflammatory and neuroprotective effects at the molecular level, the functional interpretation of SerpinA3N^+^ ASC requires consideration of their broader transcriptional context. In our dataset, the upregulation of SerpinA3N occurred in parallel with the activation of gene programs characteristic of reactive astrogliosis, suggesting that SerpinA3N^+^ ASC represent a stress-responsive reactive state rather than a uniformly protective ASC phenotype. Importantly, the expression of an anti-inflammatory molecule does not necessarily imply that the corresponding cell population exerts a net protective function *in vivo* (39). Instead, SerpinA3N^+^ ASC may constitute a heterogeneous population in which compensatory anti-inflammatory signaling coexists with broader reactive transcriptional programs induced by overwhelming inflammatory stress. Under such conditions, the protective capacity of SerpinA3N may be insufficient to counterbalance sustained neuroinflammatory cues, resulting in a reactive state that is context-dependent and potentially maladaptive if prolonged. Consistent with this interpretation, the expansion of SerpinA3N^+^ ASC was accompanied by a relative depletion of ASC subpopulations associated with homeostatic or developmental signaling programs. The loss of these homeostatic ASC functions may reduce inhibitory constraints on MG activation, thereby facilitating persistent immune activation and delaying inflammatory resolution (40, 41). Together, these findings support a model in which SerpinA3N^+^ ASC reflect a compensatory but potentially dysregulated reactive ASC state, rather than a purely neuroprotective ASC population.

The vascular compartment exhibited parallel dysregulation, highlighted by the loss of Cxcl12^+^ ECs. Cxcl12 was crucial for maintaining vascular homeostasis, and its depletion likely disrupts ECs integrity, increasing BBB permeability and amplifying neuroinflammation, consistent with the dual roles of chemokines described in cerebral ischemia (42, 43). Similarly, the reduction of Gpr34^+^ Ptgs1^+^ MG points to a loss of a specialized MG subpopulation with putative immunomodulatory and pro-angiogenic functions. Ptgs1 (COX-1) was essential for prostaglandin biosynthesis, which may influence inflammation and angiogenesis (44, 45), while Gpr34 was thought to provide inhibitory signals that restrain MG activation (46). The concurrent downregulation of these molecules points to the depletion of a MG phenotype implicated in balancing the immune response and supporting vascular repair. This depletion is observed in conjunction with an exacerbated post-ischemic environment.

Integrating these observations, we propose a conceptual model in which the post-ischemic tissue fate was shaped by the dynamic interplay between detrimental inflammatory activation and compromised reparative signaling. Notch1 signaling emerged as a pathway associated with transcriptional programs linked to tissue repair–related processes. Our data suggest that impairment of this pathway is associated with altered vascular remodeling–related and neuroprotective transcriptional programs. This was supported by recent evidence showing that Notch1 signaling can protect neurons by inhibiting the JAK2/STAT3 pathway and pyroptosis (47), highlighting its broad, cross-cell-type regulatory role (48).

Our study also introduces an interdisciplinary therapeutic framework integrating cell biology with TCM. We identified SAL as a compound associated with modulation of Notch1-related signaling pathways, aligning well with previous findings. As summarized by Jin et al., SAL exerts neuroprotective effects via coordinated regulation of inflammation, oxidative stress, apoptosis, and autophagy (49). Our data provide a cellular and molecular basis supporting these effects, indicating that SAL was associated with shifts toward ASC phenotypes characterized by transcriptional features linked to repair, potentially involving changes in Notch1-related signaling, as well as concurrent changes in the immune microenvironment consistent with a transition from pro-inflammatory toward pro-regenerative states. Huang et al. similarly reported that soybean isoflavones mitigate ischemic injury and reduce apoptosis by activating the Notch1/Hes5 pathway (50). Collectively, these studies, highlight the therapeutic promise of TCM as a multi-target, multi-level approach for stroke treatment. Notably, other herbal components may influence key cell populations such as Cxcl12 and Ptgs1 (51, 52). These findings provide a cellular framework for understanding how a bioactive compound derived from TCM may exert coordinated effects on developmental signaling and immune-related processes. This multifaceted engagement corresponds with the two core pathological processes in IS: vascular dysfunction and inflammatory dysregulation.

Despite the valuable insights provided by this study regarding cellular mechanisms and pharmacological regulation, several limitations remain. Given the exploratory nature and limited sample size of the *in vivo* validation, these findings should be interpreted as supportive rather than definitive. First, although the transcriptional features of the identified Notch1^+^ Hes5^+^ ASC subpopulation suggest a potential role in tissue repair, direct functional evidence demonstrating a causal contribution of this subpopulation to post-ischemic recovery is currently lacking. Functional validation using genetic manipulation, lineage tracing, or targeted ablation will be required to establish its necessity or sufficiency in mediating repair processes. Second, our analyses were primarily conducted in animal models, which, although informative for mechanistic exploration, may not fully recapitulate the complexity of human IS pathology. Future studies leveraging human brain tissue, single-cell atlases, or organoid systems will be essential for validating cross-species applicability. Third, although we identified key pathways and cell populations influenced by SAL, the detailed molecular mechanisms governing their interactions across different pathological stages remain incompletely understood. Moreover, clinical evidence is currently limited, and optimal dosage, therapeutic windows, and potential side effects require systematic evaluation. Addressing these gaps will be critical for advancing translational relevance.

From a translational research perspective, the ASC associated signatures identified in this study, including Notch1 and Hes5, may be amenable to future validation in human IS. These markers could potentially be evaluated in surgically obtained brain tissue samples using techniques such as immunohistochemistry, spatial transcriptomics, or single-nucleus RNA sequencing. In addition, ASC derived extracellular vesicles or cerebrospinal fluid associated biomarkers may reflect dynamic changes in glial cell states, offering minimally invasive detection pathways for clinical assessment. While these possibilities remain speculative at present, they provide a conceptual framework for advancing the current findings toward human-relevant validation.

## 5 Conclusion

This study aims to establish a single-cell-resolution analytical framework that links ischemia-associated ASC state transitions with the modulation of neuroinnate immune-vascular transcriptional networks by SAL. We identify key cellular subpopulations associated with pathological and repair-related transcriptional remodeling. A mechanistic framework centered on Notch signaling and neuroinnate immune-vascular interactions is proposed, providing a theoretical basis for developing novel recovery strategies. Furthermore, by integrating single-cell transcriptomics with pharmacological analysis of SAL, we elucidate the molecular mechanisms underlying SAL regulation of relevant signaling pathways following IS.

## Data Availability

The original contributions presented in the study are included in the article/supplementary material. Further inquiries can be directed to the corresponding authors.
